# Generating Trust in Participatory Research on *Plasmodium knowlesi* Malaria: A Study with Rural Community Gatekeepers during the COVID-19 Pandemic

**DOI:** 10.3390/ijerph192315764

**Published:** 2022-11-26

**Authors:** Nurul Athirah Naserrudin, Richard Culleton, Pauline Yong Pau Lin, Sara Elizabeth Baumann, Rozita Hod, Mohammad Saffree Jeffree, Kamruddin Ahmed, Mohd Rohaizat Hassan

**Affiliations:** 1 Department of Community Health, Faculty of Medicine, Universiti Kebangsaan Malaysia, Kuala Lumpur 56000, Malaysia; 2 Borneo Medical and Health Research Centre, Faculty of Medicine and Health Sciences, Universiti Malaysia Sabah, Kota Kinabalu 88400, Malaysia; 3 Sabah State Health Department, Ministry of Health, Kota Kinabalu 88590, Malaysia; 4 Division of Molecular Parasitology, Proteo-Science Center, Ehime University, Toon, Ehime, Matsuyama 791-0295, Japan; 5 Faculty of Social Sciences and Humanities, Universiti Malaysia Sabah, Kota Kinabalu 88400, Malaysia; 6 Department of Behavioral and Community Health Sciences, School of Public Health, University of Pittsburgh, Pittsburgh, PA 15261, USA; 7 Department of Public Health Medicine, Faculty of Medicine and Health Sciences, Universiti Malaysia Sabah, Kota Kinabalu 88400, Malaysia; 8 Department of Pathobiology and Medical Diagnostics, Faculty of Medicine and Health Sciences, Universiti Malaysia Sabah, Kota Kinabalu 88400, Malaysia

**Keywords:** *Plasmodium knowlesi*, field study, gatekeepers, exploratory study, participatory research, interview, observation, malaria prevention, challenges control

## Abstract

Background: *Plasmodium knowlesi* malaria is a zoonotic infection that affects rural communities in South East Asia. Although the epidemiology of the disease has been extensively researched, the voices of individuals within affected communities often go unheard. Here, we describe a study that explores the importance of gatekeepers in conducting research among rural communities, their perspectives on the challenges encountered when attempting to avoid malaria infection, and their views on participatory research. Methods: Between 1 November 2021 and 28 February 2022, we conducted a study in Kudat district, Sabah, using a multi-method design. All participants consented to the study, which included health care workers (HCWs) (*n* = 5), community leaders (*n* = 8), and faith leaders (*n* = 1). We conducted interviews, transect walks, and observations with gatekeepers to ensure data trustworthiness. All interviews were conducted in the Sabah Malay dialect. The sessions were audio- and video-recorded, transcribed into English and analyzed using thematic analysis. Results: Between 2017 and 2021, the number of cases of *P. knowlesi* malaria detected in humans ranged from 35 to 87 in villages under the care of the Lotong primary health care clinic. The challenges in controlling malaria include social norms, lifestyles, socioeconomic factors, environmental factors, and limitations of basic resources. Critical discussions regarding participation with the gatekeepers identified that face-to-face interviews were preferable to online discussions, and influenced willingness to participate in future research. Conclusion: This study was conducted among village gatekeepers during the COVID-19 pandemic and generated information to drive methodological changes, opening up new ideas by sharing perspectives on challenges in *P. knowlesi* malaria control among vulnerable communities. The study generated trust in the community and expanded knowledge regarding participation that is critical for future community-based studies.

## 1. Background

In the past decade, the northern region of Kudat district of Sabah has been an important study site for many *P. knowlesi* malaria studies [1]. In response to the increasing incidence of human cases of *P. knowlesi* and the attention given by the World Health Organization (WHO), many quarters have proposed the development of specific guidelines to control *P. knowlesi* zoonotic malaria [2]. Most studies have focused on the parasite and vector, while community surveys have relied on conventional research methods that assess associations and relationships between various factors and *P. knowlesi* malaria infection [3,4,5]. While quantitative studies can provide valuable insights about prevalence and scope of key health issues, qualitative studies can help to explain why people have certain thoughts and feelings that affect the way they respond to a health situation [6]. Importantly, qualitative and quantitative studies can provide data that complement each other [6]. However, most studies conducted amongst communities at risk of *P. knowlesi* malaria have collected quantitative information such as bed net usage, travel history, contact with macaques, and daily activities relevant to zoonotic malaria exposure [3,4]. Knowledge gaps remain, especially regarding the voices and perspectives of community members. A lack of sufficient knowledge and in-depth understanding of the target population and the context in which they live could negatively impact zoonotic malaria prevention and control measures.

Participatory research methodologies are recommended to obtain communities’ active involvement in exploring health issues [7,8]. The rationale behind engaging the local community in any health-related research is to the freedom to diretly express their concern, empower community members, increase the study results’ validity, and ultimately ensure broader support from the community in adopting the recommendations informed by the research outcomes [8]. They also allow interpretation of participants’ experiences regarding the phenomena of interest, and could provide the best explanations for human behaviors [6,9]. For example, in the Phillipines, the participation of communities in a photovoice study helped researchers understand the reasons for certain behaviors practiced by local communities exposed to malaria [10]. Participatory research allows researchers to bridge the gap between science and real-world practice, via community engagement that values reciprocal knowledge translation, and social action that fixes power imbalances to improve health equity [8].

Previous fieldwork has highlighted the importance of engaging gatekeepers to ensure the success of transcultural research, as they can either obstruct or facilitate the research process [11]. The gatekeeper’s role may change temporally and spatially during the research and within different organizational contexts [12]. For studies that involve minority or rural populations who often face inequities and issues of exclusion or marginalization, the gatekeepers represent the most practical access for the researchers to reach their target participants [13].

## 2. Methods

### 2.1. Study Aim

Our study aimed to describe how researchers gained access to the rural communities of Northern Sabah, Malaysia, by generating trust with the gatekeepers. The study was based on the concepts of respect towards the participants, knowledge exchange, listening to community voices, and readjusting the study methodology by incorporating community perspectives [8]. Through this study, we set out to address the following research questions:

- How important are the roles of gatekeepers among rural communities in Northern Sabah, Malaysia?
- What are gatekeeper perspectives on the challenges faced by the community related to malaria control?
- What are the perspectives of gatekeepers regarding participatory studies?

### 2.2. Study Design

This study was conducted between 1 November 2021 and 28 February 2022. This was a multi-method design study, using quantitative (QUAN) epidemiological data on *P. knowlesi* malaria incidence collected from health care workers (HCWs), and qualitative (QUAL) data obtained from transect walks, observations, and semi-structured interviews with gatekeepers. The QUAL component was the focus of this study. The COVID-19 pandemic affected how the study was conducted; for example, living with the villagers was not possible. Instead, the researchers spent time in the village from 8 am to 5 pm daily for one to two weeks a month. The study protocol has been described previously [14].

All participants gave verbal and written consent, including the use of audio and video recording. The participants were fully vaccinated against COVID-19, and social distancing and mask-wearing were practiced throughout the interviews. The interviews were conducted at the participants’ homes or their preferred locations.

### 2.3. Study Location

The study was conducted in the *P. knowlesi* malaria-endemic area Matunggong, Kudat in Sabah, Malaysia [15]. Approximately 101,700 people live in Kudat, the majority of whom are of Rungus ethnicity and Christian religion [16]. They live in hamlets that consist of several houses or longhouses [17]. The majority of the community members work as farmers [3], workers at oil palm plantations [18,19], and other forest-related employment [20]. Previous studies associated the communities of Kudat, Sabah as highly exposed to *P. knowlesi* malaria [5,19]. In Kudat, the *P. knowlesi* malaria incidence was estimated to be as high as 4.29% to 8.82% in the central eastern part of the district, where there is an expansion of oil-palm plantations surrounded by dense forest [15]. In this district, 6.2% of asymptomatic cases were detected among those who were not previously considered high risk, such as women and children below 15 years old [21]. The detection of peridomestic and asymptomatic infections highlights the threat of this zoonotic disease in the community [21]. There is a temporal relation with the monsoon season, as *P. knowlesi* malaria cases increase throughout the year after the rainy season; either during the northeast monsoon from May to September or the southwest monsoon from November to March [19].

**Figure 1:**
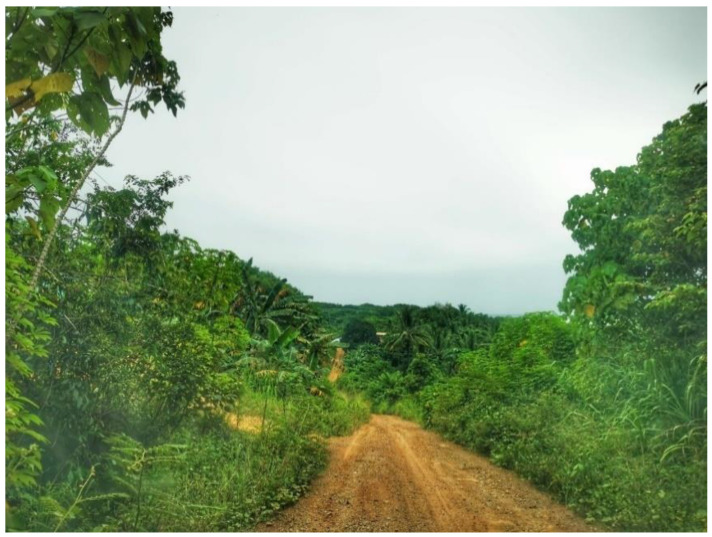
Entry route to the study area.

*Anopheles balabacensis*, the primary vector for *P. knowlesi* malaria, is present in the region. This mosquito species often bites after dark (6 p.m.) in outdoor areas [19]. Individuals who conduct outdoor activities in or near forest areas after dark are at a high risk of infection [18,19]. The route into the study area is unpaved, thus necessitating the use of a four-wheel drive (4WD) vehicle (Figure 1).

In the study area, a single rural primary health care clinic provides basic health care services to the local community. At the time of the study, the clinic was staffed by one medical officer, three medical assistants, five nurses, five officers in the malaria unit, and a driver for the ambulance and 4WD vehicle. The population in the study area receives malaria services free of charge, such as malaria screening, routine insecticide-residual spraying (IRS), insecticide-treated nets (ITNs) and long-lasting insecticide nets (LLINs), awareness campaigns, education programs [19]. Patients who are diagnosed with malaria receive free malaria treatment. The effectiveness of vector control measures are questionable as they do not address the biting habit of the vector in outdoor areas and activities that put the community members at risk of outdoor bites [19]. Furthermore, detection of *P. knowlesi* malaria cases in children and households who did not travel away from home nor conduct activities in the forest, revealed the possibility of indoor and peridomestic transmission. Thus, the use of bed nets or any conventional preventive practices are essential in this area [3].

### 2.4. Participants and Recruitment

Purposive sampling was used to select HCWs who had been working in the clinic for more than five years. They were interviewed to obtain malaria-related information in the area, such as community lifestyles, main mosquito vectors, monkey and vector behaviors, preferred breeding sites, and malaria control activities. They also provided information about the villages with a high incidence of *P. knowlesi* malaria, so suitable community leaders could be recruited for the study. Thus, community and faith leaders were recruited by purposive and snowballing techniques. The community leaders were the head of villages and the head of “*Jawatankuasa Pembangunan dan Keselamatan Kampung*” (JPKK) (English: Village safety and development committee). The head and committee members of JPKK, were selected by the community living in the respective villages. This committee is under the Ministry of Rural Development of Malaysia, which aims to ensure the village’s development and handling of community programs and projects. JPKK members also serve as mediators between community members and policymakers to inform or discuss issues related to the village [22]. Faith leaders also participated in this study. In the study sites, a person who is a faith leader could be selected as the head of JPKK (CLO2). Incentives (a t-shirt) were given to the study participants as a token of appreciation. Snacks and drinks were provided to the participants during the interview sessions (Table 1).

### 2.5. Data Collection

**Figure 2:**
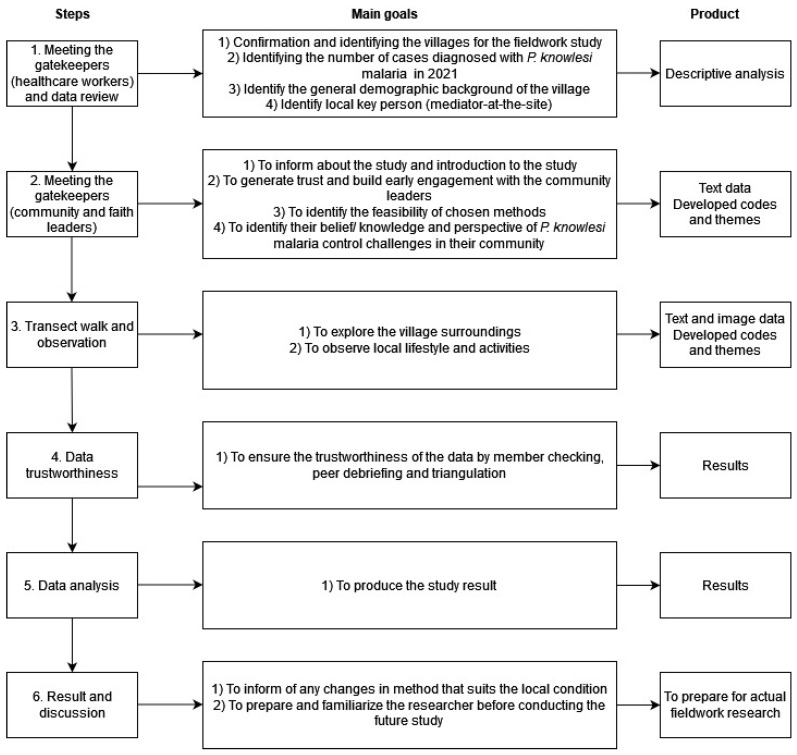
Flowchart of the systematic steps completed during the study, goals, and products.

All data were collected using an iterative process, which included data review, interviews, transect walks, and observation. The underpinning theory was adapted from the ideation model that focuses on exploring social and cognitive factors, environmental challenges, and emotion, towards intention of behaviors [23]. Using this model, Storey et al. identified factors of bed net use for malaria prevention in three African countries, such as malaria-related knowledge, awareness towards the disease and descriptive norm about its usage [24]. In this study, the model was improved by additional themes which were generated from an expert consensus using a modified Delphi study, specifically, (1) experiences of being a malaria patient, (2) feasibility of interventions, and (3) affordability of interventions [14] (Figure 2).

Step 1. Meeting the gatekeepers (health care workers)

In the initial step of the study, researchers met with the HCWs of the Lotong primary health clinic. An introductory session was conducted to clearly explain the study goals and design to the HCWs. Data review with the HCWs was conducted to identify the villages with a high incidence of human *P. knowlesi* malaria in 2021 (Supplementary File S1, Supplementary Materials).

Step 2. Meeting the gatekeepers (community and faith leaders)

**Figure 3:**
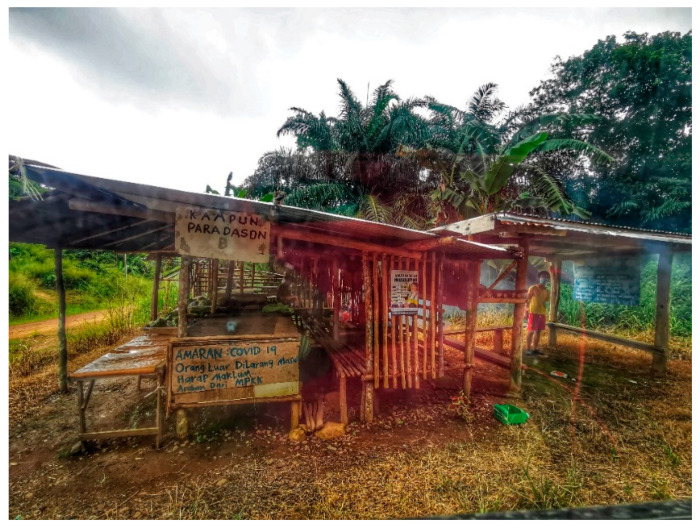
“No entry to outsiders” sign in the Malay language outside the village.

Based on the information from Step 1, gatekeeper meetings were arranged individually for the villages in Kampung Manduri (*n* = 2), Kampung Paradason (*n* = 3), Kampung Tagumamal Darat (*n* = 2), and Kampung Membatu Laut (*n* = 2); to establish early engagement and generate trust with the community and faith leaders. Community and faith leaders have the power to disseminate messages among community members and make decisions on their behalf [25]. Due to the COVID-19 pandemic, the entrance to the village was limited to outsiders and subjected to the permission of the gatekeepers (Figure 3).

During the meetings, the researcher introduced the study topic to the gatekeepers and provided relevant information. HCWs accompanied the researcher during the meetups as local translators. During this phase, the researcher played an outsider role and provided a clear explanation to the community leaders to avoid any over-expectations. The duration of each meetup was approximately 40 to 60 min. The conversation was video and audio-recorded, and all participants provided verbal and written consent. They were informed about future interviews, as they represented information-rich participants with vital posts in the communities [26].

Step 3. Transect walk and observation

Transect walks and observation were performed with the gatekeepers to develop a deeper understanding of the study site surroundings and to understand the day-to-day activities of community members [27]. This systematic walk was lead by the gatekeepers along the path to allow researchers gained a visual observation of the villages [27]. During this time, researchers had the opportunity to engage in informal conversations with the gatekeepers. Various areas were visited, including those with common monkey sightings. The transects walks were performed between 0900 and 1200 h in two of the villages (Kampung Manduri and Kampung Membatu Laut). Observations were carried out from a 4WD vehicle in the other two villages (Kampung Tagumamal Darat and Kampung Paradason) due to the rainy season and for the safety of the female researcher in the forest and plantation areas. Field notes were made, observations were recorded, including sketching of transect maps for future reference [27]. The data were triangulated with the data collected from Steps 1 and 2.

Step 4. Data trustworthiness

Data triangulation was performed using methods to enhance data credibility; including interviews, observations, and transect walks. The quality of the collected and analyzed data was enhanced by experts in the research team having different methodological, epistemological, and experiential backgrounds [6]. To ensure data rigor and credibility, member checking was performed after each step [28]; and an external expert translator fluent in the Rungus language was recruited. Field notes taken during the previous steps ensured the transferability of the study data; while confirmability and dependability were generated through quotes from the interview transcripts [28]. The co-authors played roles as additional coders apart from the first author, to increase the richness of data [29]. A robust description of the data can enhance data transferability and rigor [28], in addition to facilitating contextual evaluation [30]. Misinterpretations of facial expressions due to mask-wearing during the interviews were minimized by member checking after the interviews. These approaches were conducted to ensure the data’s trustworthiness and reduce any researcher bias [28].

Step 5. Data analysis

All field notes were transferred to a computer and stored in password-protected devices. The data collected in the study were analyzed using deductive and inductive approaches and reflexive thematic analysis [29]. Transcription of the interviews was completed by N.A.N. Next, N.A.N. iteratively read the data in-depth to familiarize themselves with the dataset. N.A.N. actively generated codes and themes from the transcripts. M.R.H. and R.H. also facilitated the generation of codes and themes to ensure the richness of the information from the raw dataset [29]. The themes were reviewed and agreed upon by the coders. The codes and themes were also generated inductively based on the researchers’ reflexivity. The strength of the data analysis lies in the meaning-making process through the subjectivity and reflexivity of the researchers [29]. Illustrative quotes were included in the study results to demonstrate key findings of the study.

The interviews and other methods in Steps 1 to 3 were conducted in Sabah Malay dialect. The quotes were translated from the Sabah Malay dialect to the English language by authors who understand the dialect and are fluent in English (N.A.N., M.R.H., and P.Y.P.L). Where a local indigenous language was used, an expert translator facilitated the translation. The qualitative data analysis was facilitated by ATLAS.ti Windows (Version 9.1.7.0).

## 3. Results

### 3.1. General Characteristics of the Study Sites

**Figure 4:**
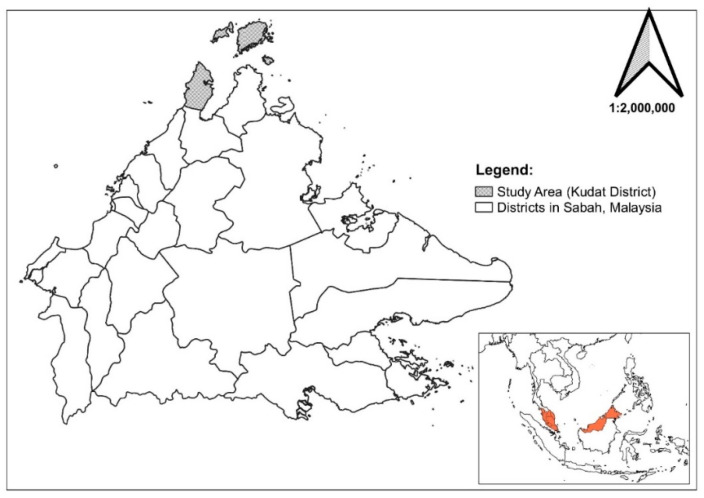
Map of the Kudat district.

**Figure 5:**
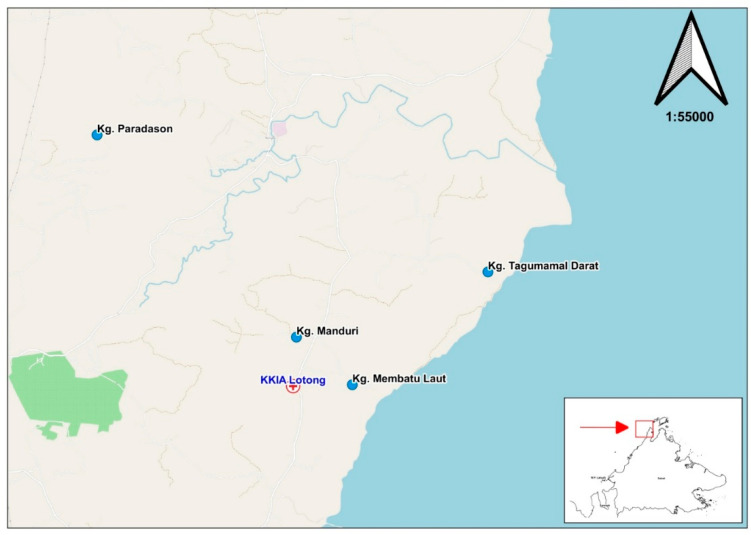
The study sites.

Surveillance data showed a continuous incidence of *P. knowlesi* malaria in the area, from 75 cases in 2017, 84 cases in 2018, 31 cases in 2019, and 36 cases in 2020. Despite the COVID-19 pandemic there were 87 cases detected in 2021. From the data, 25 of the 32 villages recorded a high incidence of *P. knowlesi* malaria cases, defined as an incidence rate of >1 per 1000 population [10]. There has been no history of fatalities or human malaria cases (namely *P. falciparum, P. vivax nor P. ovale)* in the area for a decade. Due to time and budget constraints, four villages with the highest incidence (per 1000 population) of *P. knowlesi* malaria in the year 2021 were selected and visited; Kampung Manduri (35.46/1000 population), Kampung Tagumamal Darat (72.72 per 1000 population), Kampung Paradason (169.49/1000 population) and Kampung Membatu Laut (53.76/1000 population). In the year 2021, Kampung Manduri and Kampung Tagumamal Darat had one *P. knowlesi* malaria case, detected in children below 12 years old, for each of the village, while two cases was detected in Kampug Paradason (Supplementary File S1, Table 2, Figure 4 and Figure 5).

### 3.2. Challenges to Malaria Prevention

**Figure 6:**
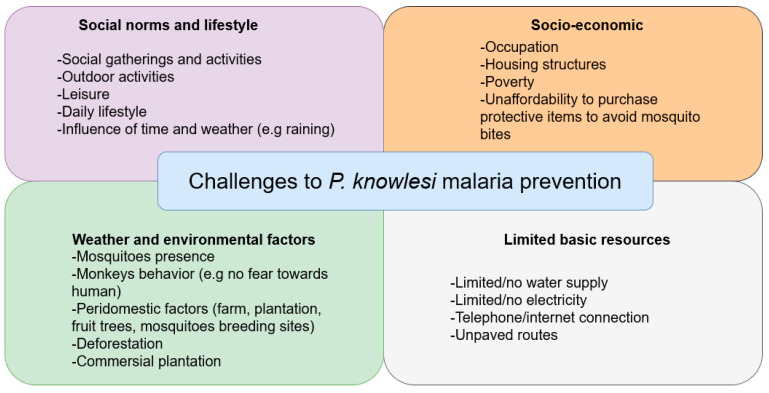
Challenges to avoid malaria infection based on the perspective of gatekeepers.

We identified factors that might serve as challenges to malaria prevention based on the perspectives of gatekeepers. The gatekeepers listed several factors that affected the possibility of getting mosquito bites and being exposed to malaria infection. For the gatekeepers, the challenges can be categorized as: (i) social norms and lifestyle, (ii) socioeconomic factors, (iii) weather and environmental factors, and (iv) limited basic resources. These factors were also viewed as contributing factors to the high incidence of “*malaria kara*”, “*demam malaria”*, or simply “*malaria*” cases, terms used in the village when they described monkey malaria (Figure 6).

### 3.3. Social Norms and Lifestyle

Community activities were regularly conducted within housing compounds, farms, forests, and plantations. The lifestyles of community members encompassed family-related activities such as conversations with neighbours, gardening, walking into the forest, fishing, hunting, and bee keeping. The lifestyles were generally influenced by activities in the village and forest-related undertakings, as travelling outside was either inconvenient due to the road conditions, unavailability of transport, or cost. Weather and time served as barriers and drivers for these activities; for example, during the rainy season, community members were unable to go rubber tapping, while the timing for rubber tapping was based on individual preferences. Hunting was occasionally done during dark hours prior to dawn. *“Aramaiti”* or alcohol drinking is a norm during festive activities and celebratory gatherings; and traditional events such as weddings continue from morning through the night, and sometimes for more than one day. Social norms and lifestyle activities that are mostly performed outdoors and during night-time increase the risk of exposure to mosquito bites (Supplementary File S2, Supplementary Materials).

*“The community here is mostly farmers who work in the forest. Like me, I also have my farm and I plant vegetables, go fishing, or look for honey in the forest. Here, it is common for people to gather and drink. We called it “Aramaiti”. Maybe mosquitoes bite them when they fell asleep outside their houses. The villagers like to take showers outside their homes and hang around in the late evening or even at night. And do you know? The rubber tappers are still outside in the evening. Sometimes around 5 o’clock to 7 o’clock, or even 9 o’clock. Only when they feel satisfied working on the whole farm, then they will return home.” [HC03]*

During transect walks and observations, it was perceived that most of the houses were built with rattan and bamboo. Most houses have structures that allow for mosquito access due to holes and cracks, and mosquito nets were not generally present on doors and windows. When asked about housing structures, gatekeepers commented that tradition influenced the structure of the house; for example, the importance of “tinkang” (English: verandah), and a toilet or bathroom unattached to the main housing structure was evident. Thus, traditional architecture could pose challenges in the prevention of mosquito bites. During our observations, we found a modern house with a “tingkang” that was built as an extension of the original house structure. One community leader discussed how it is used:

*“We like to sit at the “tinkang”. Do you know what is “tinkang”?. Rungus people like to build “tingkang” at home. We will sit, chit chat, and even sleep there (pointing to the verandah). Usually, there will be an area within the Rungus house that is not an enclosed space. Before sleeping around 7–9 pm, we sometimes sit there and chit chat. This area is called “tingkang”. Because it is an open space, mosquitoes can fly in and out easily.” [CLO2]*

### 3.4. Environmental Factors

**Figure 7:**
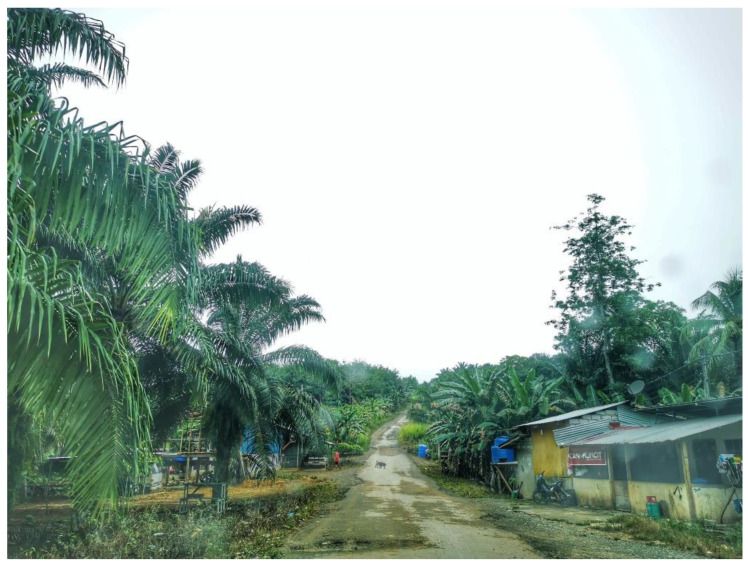
Commercial oil palm plantation (left), with coconut and banana trees at the village.

**Figure 8:**
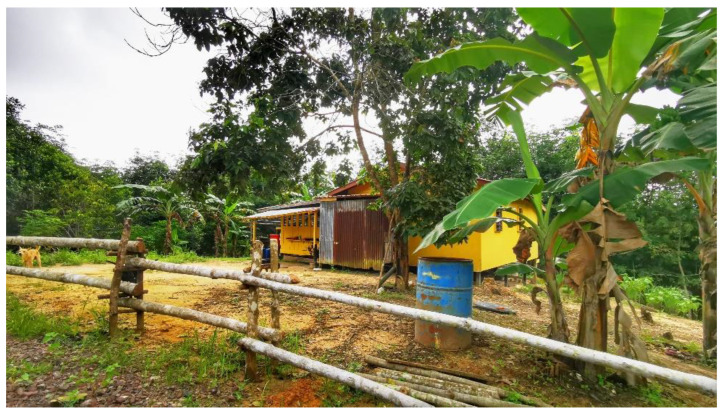
A local villager’s house surrounded by forest, plantation and fruit trees.

The study sites were located in areas surrounded by forests and hills. There was a mix of plantation areas such as commercial rubber and oil palm plantations, and family-owned banana and coconut farms (Figure 7). Houses were built individually on family land, with rubber and oil palm planted nearby (Figure 8). Fruit trees such as banana, mango, and papaya were also seen, and were an attraction for monkeys. Based on gatekeepers’ information, long-tailed monkeys are regularly seen in troops of 10 to 50. According to gatekeepers, the behavior of monkeys was concerning, as they were described to show no fear towards humans and destroyed their crops, plantation, and fruit trees. Water pools and stagnant water were found on the grounds, and possibly in tree holes. These are potential mosquito breeding sites and therefore a concern for malaria intervention (Supplementary File S2, Supplementary Materials).

*“Ten years ago, there were fewer monkeys here. Human activities might be why the monkeys and mosquitoes are getting closer to humans… trees were cut down… there are less forest areas now… [paused]. In our village, we are surrounded by oil palms, rubber trees, coconut trees… banana… and monkeys… there are everywhere! [They are] destroying the fruit trees, crops and plantations… [CL08]*

### 3.5. Socioeconomic Factors

Socioeconomic factors such as daily occupational-related activities and housing structures are related to exposure to mosquito bites and make it challenging to control malaria in the village. Activities such as farming, fishing, collecting forest plants, vegetables, and honey, and forest-related work increase the risk of mosquito exposure. The majority of the villagers work as farmers, rubber tappers, and oil palm workers. During these activities, the gatekeepers expressed that practices to avoid mosquito bites were not sufficiently performed due to several individual reasons. Poverty and the inability to purchase mosquito repellent, for example, increase the risk of mosquito bites as these occupational activities were not performed at any specific time. Due to poverty, houses were traditionally built using materials from the forest (Supplementary File S2, Supplementary Materials).

*“Communities here, their lifestyles are all related to the forest and their farm… They go fishing, plant vegetables, and walk to the rubber farm. Do you know that these works can be done anytime? …. In the morning, even during night time… early morning too! People can go to their rubber farms even at night! This is how they get money for their families…When the weather is good, what I mean by good is when it is not raining…they can get more “kantalan (English: thick rubber sheets).” [HC01]*

### 3.6. Limited Basic Resources

**Figure 9:**
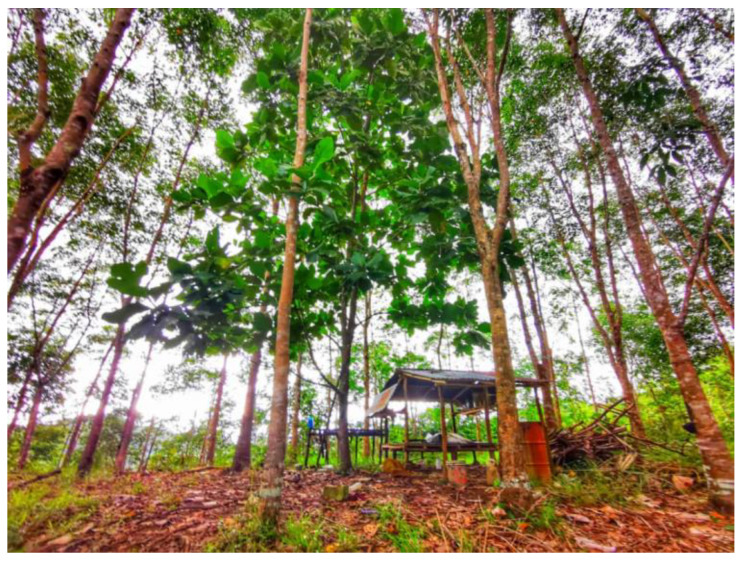
The “sulap”.

Limited basic resources such as water and electricity supply, telephone and internet lines, as well as road connections were common in the villages. For example, the village roads were not paved and the roads were occasionally blocked due to erosion during heavy rains. Villagers faced frequent interruptions to clean water supplies and poor coverage of internet connections. During the pandemic, when school classes were conducted online, children were exposed to mosquito bites as they needed to climb hills to connect to the internet. Those who require online access are exposed to a similar risk. In certain areas, “*sulap*” or small huts were built to serve as multipurpose buildings for rest, online activities, and sleeping after farming activities (Figure 9). Based on information from gatekeepers, the river is their water source for laundry, bathing, and washing dishes. These limited resources affect malaria control efforts, as they increase human contact with mosquito bites.

*“Do you know that we often have water supply issues?…It is difficult for the villagers as they need to go to the river to clean themselves, wash clothes, and do other chores.” [HC04]*

### 3.7. Gatekeeper’s Perspective on Participatory Research

Gatekeepers reported that face-to-face interviews are more feasible than online interviews. Some of the community members might live in hard-to-access areas and have personal limitations such as socioeconomic constraints prohibiting them from attending focus group discussions (FGDs). Potential participants who lived in a *sulap* far away from the main community might not be accessible for a study due to the inconvenience to both the researcher and participants (e.g., weather and safety issues). While methods were preferably skewed toward those easiest for prospective participants, gatekeepers expressed interest in participatory research and shared their concerns regarding previous studies done in their village.

*“I think focus group discussions are suitable, provided the time and place are easy for the villagers. They are farmers or rubber tappers… It might be not easy to gather them in the same place at the same time. Some will go to their rubber plantation in the morning. It also depends on the weather… If it rains, they cannot go to work. Maybe you can create a Whatsapp group to make it easier to communicate with the participants… though internet connection might be a problem… and not everyone would be able to reply urgently.” [HC01]*

With regard to participatory research, the gatekeepers showed interest as they believed that community participation would enable their perspectives and concerns to be heard and translated into action by policymakers. In addition, the study findings can be disseminated through various platforms. Future programs or studies could include community members to be stakeholders as they could bring valuable information to health programs [8,31]. The empowerment of community members can also facilitate behavioral change among other community members [32,33]. There is a need to integrate a different perspectives in disease control programs, but the success depends on the balance of the social, economic, political and environment [31] (Supplementary File S3, Supplementary Materials).

### 3.8. Reflections of the Researcher

#### 3.8.1. Positionality: Insider-Outsider Role

Steps 1 through 3 allowed the researchers to become familiar with the study settings, and reflect on the positionality of the outsider–insider role in research. During the study, the researcher positioned themselves as an outsider when performing early engagement with the gatekeepers, due to the cultural differences with the study population. However, from our perspective, as we gained access to the villages by the end of the study, the role transformed from being an outsider to an insider. The gatekeepers opened up and shared their perspectives. As the continuum of participatory research ranges from minimal involvement to full participation of community members as co-researchers [8], the role of researchers in this study are situated between the two corners. The researcher influences the overall aim of the study and the future recommendation, while the gatekeepers play their role in supporting the work, advising, and sharing their perspectives through the interviews. Total participation was not adapted in this study given the possible issues such as limitation of funding, possible opportunistic situations, and to minimize separation between the community, the researcher and other larger groups (e.g., policymakers) [34]. Through the engagement practiced throughout the study, the community leaders agreed to participate in future interviews related to the study:

The researcher: *“I will come again later to interview you about your perspective on monkey malaria.”*

*“You are most welcome. Please inform me earlier so I can adjust my timing and prepare myself for the interview!” [CL04]*

From the study, we learned not to interfere in cultural practices or death events and to respect the privacy of all individuals in the households at all times, including having a female chaperone when interviewing any women in the village households. As outsiders, we tried to be considerate and not encroach on private domains such as kitchens and bedrooms, unless we were given permission to enter the “private” area. To minimize possible embarrassment and conflict, we decided that sensitive questions such as family incomes will not be asked directly. We opted for indirect interview questions such as “*How do you routinely use insect repellent*” or “*How affordable is it for you to purchase long pants and shirts for your whole family members?”.* Recognizing “sensitive” issues and being aware of their responses and our words are essential to becoming the “insider” in the study settings.

#### 3.8.2. Trust

In generating trust among the gatekeepers, we tried to be socially and culturally aware and thoughtful with our communication and attitudes, and to avoid mistrust, research misconduct, and ethical issues. We highlight several motivating factors that can facilitate future participatory research studies:

1. Consent: The gatekeepers signed a consent form to participate in the study.
2. Gatekeepers: The community leaders granted permission to the researchers to conduct the study in their villages during the COVID-19 pandemic. They volunteered to mediate access to prospective participants. To facilitate communication, they shared their phone numbers with the researchers.
3. Language and translators: The Sabah Malay dialect was used as the main language, while some gatekeepers became local translators for the Rungus language.
4. Trustworthiness of the data: The raw data and analysis were shared with the gatekeepers to ensure credibility through member checking and correcting any misinterpretations made by the researchers.
5. Social justice: The researchers included the gatekeepers’ perspectives to strengthen the study findings and views during the participatory study.

#### 3.8.3. Methodological Concerns

When planning for the study, to minimize interruptions, the timing of research activities should be arranged based on the daily schedules of the participants. For example, several planned visits to the gatekeepers were unsuccessful as they were not available. Efforts should be made to compensate for the participant’s time with appropriate tokens (e.g., foodstuffs) since they have to forego other economically-productive activities during the time that they participate in the study.

The preferences of community members must be considered regarding which aspects of data collection they wish to participate. The researchers must be aware of their preferences as they can influence the study process. To achieve high participation and high quality data, the most appropriate method feasible for the participants should be selected to minimize conflicts between researchers and participants [35].

At times, while we were focused on gathering data related to the research objectives, we tended to neglect other priorities and needs discussed by the local community, such as the lack of necessities, including electricity and water supply. The behavior of monkeys that encroach on their plantations will be the focus of future studies. Despite not being directly related to the research objectives, these issues should not be ignored. Acknowledging concerns can enhance the rapport with the participants.

Researchers should try to adapt to the social surroundings by adopting simple clothing and appearance to improve rapport with the local people. Layman terms should be used during interviews, such as greeting the participants in the local language; for example, *Salamat sarap*, which means good morning. Medical jargon should be avoided, such as the scientific term “*Plasmodium knowlesi*” in favor of the term “*Malaria kara*”, which refers to monkey/zoonotic malaria. There were times when the participants had difficulties understanding questions placed on them, and thus the role of a local translator is essential. The community might be unfamiliar with our terminology and misunderstood the questions. Thus, some flexibility should be practiced when asking questions during interviews. Informal conversation can lead to more success in obtaining the data needed [35]. In addition, we noticed that certain conflicts such as political situations and workloads within the communities might influence the research process. Thus, it is vital to maintain a neutral stance. We must also stress to the participants that we are merely interested in their opinion, knowledge, and beliefs. There is no right or wrong answer, and all information will be confidential.

## 4. Discussion

To the best of our knowledge, this is the first study to explore the roles of gatekeepers in rural communities in Sabah, Malaysia and to evaluate their perspectives on the challenges of avoiding malaria. We also explored their views on participatory research. Despite the challenges faced during the COVID-19 pandemic, the study generated new knowledge and expanded our understanding of various factors influencing *P. knowlesi* malaria prevention. It allowed us to generate contacts with gatekeepers and establish trust, both of which are essential for participatory research [36]. It facilitated closer relationships between the researcher, gatekeepers, and prospective participants. 

*P. knowlesi* malaria poses a challenge for the malaria elimination programme because the transmission involves a natural reservoir, the Macaque *fascicularis* and *M. nemestrina* monkeys. These natural hosts move closer to human settlements, largely attributed to anthropogenic activities such as deforestation [37,38]. The behavior of the anopheline mosquito vector, from the *Leucosphyrus* group is exophilic, and feeds at dusk and dark [39]. While previous studies have established the essential roles of the behavior of monkeys and mosquitoes, it is critical to acknowledge the role of human behavior in the risk of *P. knowlesi* malaria [40]. Human behaviors are complex and are influenced by multiple factors such as social norms, attitudes, and social constructs [41]. Thus, at the community level, understanding the beliefs, perspectives on the disease, and challenges to avoiding mosquito bites can inform the development of appropriate and sustainable disease control interventions [41]. In future studies, the participation of these gatekeepers and other participants can facilitate the design of locally-customized tools for *P. knowlesi* malaria control. Collaboration between multiple agencies and communities at risk is needed for future research and policy design concerning vector-borne diseases [25]. Specifically, for *P. knowlesi* malaria control, the presence of strategies that incorporate multisectoral collaborations should be strengthened to minimize the risk of disease among vulnerable communities [42]. 

This study highlights the positive role of the creation of early engagement with the gatekeepers in participatory research. It is vital to obtain trust among the rural communities as fieldwork research requires negotiation and manoeuvring into the best position for data collection. It also involves the context of unequal power relations while maintaining professional and ethical conduct [43]. Researchers should continuously reflect on the outsider–insider role in conducting research among rural communities. The ability to gain access and conduct studies at a time when no outsiders were allowed to enter the villages during the COVID-19 pandemic demonstrated that trust is a crucial element that can arise through the process of conducting participatory research, particularly across cultures [44]. Thus, it is vital for researchers to understand and respect local contexts, cultures, and attitudes. However, continuous trust needs to be maintained as gatekeepers are influenced and driven by different priorities which could affect the research project’s outcomes [45]. Therefore, to guarantee their continuous participation, researchers should iteratively reflect during the study steps; for example, by reinforcing a respect of community perspectives, adjusting research methodologies to make it flexible for participants [8], and minimizing conflicts [44]. Researchers must continue to engage with the participants to understand their life challenges, and explore their social and health inequities [8].

In this study, we highlight the important relationship between researchers and gatekeepers in the field, as they are the point of entry and can support the success of a study progress [12]. Previous research has shown that the involvement of community members represents the main component of a successful malaria strategy [25]. The sustainability of malaria interventions must take into account the local social context and behaviors of the community members [41]. In adressing this issue, qualitative studies have the strength to enable researchers to understand the logic behind human behaviors [46]. This study provides guidelines and considerations for future researchers who are interested in conducting research with rural communities.

The COVID-19 pandemic influenced our study methods. Effective communication is essential to minimize the risk of malaria during COVID-19 pandemic [47]. We conducted our study with gatekeepers to minimize possible misconceptions and rumours that can affect the local perspective of malaria prevention. Despite the pandemic, the vulnerable population exposed to malaria must be made aware that the risk for malaria infection remains high despite COVID-19. For example, they should seek medical attention within 24 h of fever onset [47]. Meeting the gatekeepers and becoming familiar with them was essential before conducting participatory research [13]. The gatekeepers have a right to know the proposed research steps or phases and the potential impact on their communities. People who live in malaria-endemic areas may consider malaria as an ordinary event that does not pose a threat to them, thus thinking that they need not practice preventive measures or seek appropriate treatment [48]. Future *P. knowlesi* malaria studies should consider possible transformation in disease control strategies from social science perspectives that focus on informal practices in people’s daily lives, and the challenges they face due to their environmental, socioeconomic, and limited basic resources.

From the public health point of view, community participation in research should be viewed as an essential goal to achieve social justice and collaboration with the local community. Public health researchers can maximize their impact in performing their responsibility to promote social justice by partnering with the community to be vocal about their experiences and emphasizing the importance of community power to make life changes [8]. Engaging with the community in dialogue sessions enables them to reflect on their values and strengthen their relationships with one another so that they can be empowered to make healthy choices [8]. Importantly, the community is in a unique position to best describe the conditions and resulting challenges from their own points of view [49]. While we acknowledge that previous research in this area has made a dynamic contribution, more evidence in the form of reflections from researchers and community members engaging in participatory approaches to address malaria risk is needed.

Despite the COVID-19 pandemic, we managed to engage with the gatekeepers. The development of such relationships can strengthen the essence of participatory research. When assistance or advice is needed in the future, these gatekeepers can be called upon as allies [36]. The quality of human relationships forged in the field will determine the success of research compared to just faxing or emailing the study proposal to the organization [36] Moreover, maintaining the right attitude and taking the opportunities to engage with the local community can boost the confidence of the community members toward the researchers. Studies in the field can determine the success or failure of a research project [50]. Some problematic methods in the initial planning, seemingly dead-ends, were modified after obtaining feedback from the gatekeepers. The steps taken in the study can reduce the likelihood of failure in future studies. While *P. knowlesi* malaria prevention and control remains a challenge, researchers must pay attention to social challenges. Meaningful community involvement in research studies can facilitate public health researchers in addressing these issues and break the cycles of social and health inequity and other challenges related to disease control.

### 4.1. Strengths of the Study

- Extensive field visits and interaction with the gatekeepers generated powerful knowledge, information, and trust that can support future studies.
- An equity lens was central to this study, to ensure that the gatekeepers could disseminate vital messages to community members.
- The study generated an understanding of social and health inequities among the study communities.
- The selection of the study sites built upon previous study findings and the understanding of social and environmental factors influencing *P. knowlesi* malaria exposure.
- Active participation with gatekeepers was achieved by modifying existing methodologies by considering lifestyle and activities of the involved communities; thus currently, the researcher has gained access and started follow-on participatory research at the study sites.

### 4.2. Limitations of the Study

- We could not conduct an ethnography study, to fully immerse ourselves in the local languages and ways of life, due to the COVID-19 pandemic.
- Costly resources were used in this study; for example, 4WD vehicles and drivers to travel to study areas inaccessible by sedan cars.
- The study was time-consuming as the researcher had to conduct negotiation sessions with the gatekeepers and iterative field visits to establish trust over time.

## 5. Conclusions

This study emphasizes the benefits of engaging with gatekeepers in a community exposed to *P. knowlesi* malaria on Northern Borneo Island and the challenges of conducting fieldwork during the COVID-19 pandemic. Our findings suggest that gatekeepers play important roles and have the ability to make decisions on behalf of community members, which are essential for open communication, and facilitation of future research, health advocacy, and the management of village resources. Gaining gatekeepers’ perspectives helped researchers to understand the multi-level influences of social norms, lifestyles, culture, socioeconomic status, and environment on malaria control initiatives. The study revealed issues with limited basic resources, and the influence of weather and time, which expanded our knowledge of the barriers faced in avoiding malaria. The gatekeepers recommended ways to adjust the proposed research methodology based on their experiences, and were positive regarding the prospects of participating in future research. Early engagement with gatekeepers in building trust is vital to create a platform for conducting future studies and projects in the community. Lastly, the study provided important information about social issues related to disease exposure in its natural setting, allowed recognition of social and health inequities, and provided a foundation for the team for conducting research with the local communities in the future.

## Figures and Tables

**Table 1:** Characteristics of study participants.

Gatekeepers	Age	Gender	Ethnicity	Occupation	Years of Working as Health Care Workers	History of Malaria Infection
HC01	48	Male	Rungus	Malaria officer	15	Once in 2021
HC02	53	Male	Rungus	Malaria officer	30	Five times, twice during childhood, the most recent in 2020
HC03	44	Male	Rungus	Malaria officer	17	Once during childhood
HC04	37	Female	Rungus	Staff nurse	12	Unknown
HC05	36	Female	Rungus	Staff nurse	12	Once in 2016
CL01	62	Male	Rungus	Farmer	-	Yes
CL02	55	Female	Rungus	Housewife	-	No
CL03	62	Male	Rungus	Farmer	-	Two to three times every year, the most recent in 2017
CL04	46	Male	Rungus	Farmer	-	2020
CL05	40	Male	Rungus	Farmer	-	No
CL06	38	Male	Rungus	Farmer	-	Unknown
CL07	61	Male	Rungus	Farmer	-	Yes
CL08	67	Male	Rungus	Ex-Government officer	-	Yes
CL09	45	Male	Rungus	Farmer	-	Yes

**Table 2:** Characteristics of the study villages.

Study Sites	Kampung Manduri	Kampung Paradason	Kampung Tagumamal Darat	Kampung Membatu Laut
Total population	141	59	167	164
**Age group**				
Children (<18)	49	10	46	54
Adult (>18)	82	37	101	100
Elderly (>65)	10	12	20	10
**Gender**				
Male	79	30	86	70
Female	62	29	81	94
Ethnicity	Rungus (100%)	Rungus (94%)	Rungus (99%)	Rungus (94%)
		Dusun (4%)	Dusun (1%)	Dusun (5%)
		Tabilong (2%)		Kimaragang (1%)
Religion	Christian (98%)	Christian (95%)	Christian (100%)	Christian (98%)
	Islam (2%)	Islam (5%)		Islam (2%)

## Data Availability

The qualitative verbatim relevant to the study in the Atlas.ti database is available from the corresponding author upon reasonable request.

## Supplementary Materials

The following supporting information can be downloaded at: https://www.mdpi.com/article/10.3390/ijerph192315764/s1.
