# Supplementary material for: Generating Trust in Participatory Research on Plasmodium knowlesi Malaria: A Study with Rural Community Gatekeepers during the COVID-19 Pandemic
Source: Int J Environ Res Public Health. 2022 Nov 26;19(23):15764. doi: 10.3390/ijerph192315764 (PMC9737837; doi:10.3390/ijerph192315764)
Supplement: Supplementary file 1 [file ijerph-19-15764-s001.zip › Supplementary File S2. The themes and quotes.pdf]

Supplementary File S2: The themes and quotes from participants

| Themes | The quotes from participants |
|---|---|
| Social norms and lifestyle | *"Kids and adolescents will play outside their house in the evening. One of our patients got malaria because he played with his handphone until late at night outside the house. Sometimes, they hike the hill, just to get the internet lines. Kids nowadays, they like to hang outside the house..moreover, most of the toilets are built away from the house. Usually, after the villagers return from their farms in the forest, they will take a shower at the toilet outside their house. Even though 90% of the village houses have water supply, they still like to take shower outside their homes. Sometimes, they will go to take their showers outside at 7 pm!".*[HC01]<br><br>*"Previously, I was always admitted to the hospital for malaria. Since I changed my lifestyle and working hours four years ago, I was diagnosed with malaria again. I always explain this to my community members, but I think it is difficult for them. [paused] They need to go to their farm or rubber plantation to perform many activities. It also depends on the weather. If it is raining, they stay indoors. If there is no rain, they will go out to work or go to the forest. For rubber tappers, it really depends on the weather. Night time is the most suitable time for them as the rubber can flow nicely for them to collect the "kantalan" and sell that for money."* [CLO3]. "*Kantalan*"is the term used to describe the latex.<br><br>"*What makes them go into the forest? There are many plants and vegetables there. We can also catch fish in the rivers there... Do you know "menyizud" or "Sisizud"? We use that to catch small prawns and fish. We use a lot of different tools when we go fishing*. *We go anytime, in the morning, evening, and even at night. Also, there is a traditional practitioner who is around 70 years old. He is very old and he is known to be able to* |

| | |
|---|---|
| | *use plants to cure. He lives there (pointing out far), not in this village, but people will go to him to buy remedies to cure their sickness!" [*HC02].<br><br>*"It is common here…the Rungus people houses were built with rattan, bamboo, and trees. The toilets are outside. It is not practical to build an attached toilet in the house. It is just our culture. When they need to use the bathroom, they walk out of the house even at night."* [HC03] |
| Environmental factors | *"BUARAU! BUARAU! [shouted] We will shoo them away, but they only look at us and they are not afraid of us anymore. Do you know? There was a person who was taking his bath in the river. He was naked the monkey came and took his shirt? Previously, people hunt monkeys as their food…But some time ago, we were told not to kill them for different reasons…research, animal rights, many reasons…Nowadays, I rarely hear people saying they will kill the monkey because the monkeys look like humans and that it is pitiful to kill them!" [CL04]* |
| Socioeconomic factors | *"In our village…there are a lot of rubber trees, rubber trees...and mosquito bites! The villagers they will bring the mosquito repellent coil….to avoid the mosquito …some will use the mosquito incense… that will produce a stronger smell... the mosquito does not like the strong smell... but it does not last long… so they need to continuously burnt the new sticks [paused]…these items some uses them regularly, while some does not, due to cost. It is unaffordable. It is expensive!"* [CL04] |
| Limited basic resources | *"We always have problems with the internet line here…. This can be the reason for exposure to mosquito bites. The people will climb the hills or go to certain spots where internet connection is good. We will* |

| | |
|---|---|
| | *build the "sulap" in these areas…. the internet connection is not available at all places. People will commonly hang around outside their houses to play games or use Facebook. Recently, a teenager was infected with malaria… I believe he got the mosquito bites when he was using his phone for online activities, outside his house"* [HC01]<br><br>*"There is always a water problem here. For me, there is not much issue. I can go to the shop and buy water. But not everyone here can afford to do so. So, they need to go to the river to get the water to clean themselves, do laundry, and other things. Now you see, at the river, there were many mosquitoes, even monkeys! [silence]… For example, in January 2022, we didn't have water for one month! During the rainy season, we can rely on water catchment areas. If it is the draught season, where else can we get water? The river will be our only source!"* [CL04]<br><br>*"In our village, if there is no water supply, we use gravity water. Do you know during December last year, we don't have water for the three months! …We collect money from the families that are willing to share to build the water pipes. Once it is completed, the water will be pumped into their houses."* [CL09] |
