# Supplementary material for: Generating Trust in Participatory Research on Plasmodium knowlesi Malaria: A Study with Rural Community Gatekeepers during the COVID-19 Pandemic
Source: Int J Environ Res Public Health. 2022 Nov 26;19(23):15764. doi: 10.3390/ijerph192315764 (PMC9737837; doi:10.3390/ijerph192315764)
Supplement: Supplementary file 1 [file ijerph-19-15764-s001.zip › Supplementary File S3. The gatekeepers perspectives.pdf]

Supplementary File S3 The gatekeepers perspectives on participatory research

	“*This will be a great project! You can get in-depth with the community here, learn about their lifestyle, activities, and listen to their stories! But... I have never heard about Photovoice… I am excited and eager for these communities, moreover to see the result!”* [HC02]<br><br>*“The previous malaria researchers, I talked to them…. I asked them, why don’t you or we capture the monkeys and kill them? We cannot do that! They replied… These monkeys, they are destroying everything, everything (shouting]… our fruit trees like bananas, mangoes, papaya, everything! Even our paddy. I think their behaviour changed, previously they were afraid of humans but nowadays, they come into our house and disturb everything, our kitchen, our TV, radio…”* [*CL03*]<br><br>*“This kind of study can provide information on the factors that contributed to the malaria cases in our village. The ‘togetherness in conducting the study’, I hope can finally provide a way to control the malaria transmission in our village.* “[CL06]<br><br>*“Thank you for conducting this kind of study in our village! We can improve our understanding of how we can be exposed to the infection, how to avoid the mosquito bites, and find ways to reduce the number of cases… I like the Photovoice project, I like how the study will be done with the community…. But for the interview, I think face-to-face will be easier. I doubt they are able to come, even at a fixed time and place…” [CL02*]

	"I hope this study can empower the villagers by increasing their awareness to keep our villages and the surrounding areas of the houses clean. It is important for them to be aware of the breeding sites of the mosquitoes and the importance of cleaning the breeding sites." [CL04].
